# Molecular characterization of circulating infectious bursal disease viruses in chickens from different Egyptian governorates during 2023

**DOI:** 10.1186/s12985-024-02559-9

**Published:** 2024-11-30

**Authors:** Amr H. Abd El-Fatah, Dalia Ayman, Mahmoud Samir, Soad Eid, Mahmoud Elgamal, A. A. El-sanousi, Mahmoud Ibrahim, M. AlKhazindar, M. M. Ali, Amira Afify

**Affiliations:** 1 Egyptian Company for Biological & Pharmaceutical Industries (Vaccine Valley), 6th October City, Giza, 12511 Egypt; 2 Laboratory for Veterinary Quality Control On Poultry Production, Animal Health Research Institute, Agricultural Research Center, Dokki, Giza, 12618 Egypt; 3 Department of Virology, Faculty of Veterinary Medicine, Cairo University, Giza, Egypt; 4 Department of Birds and Rabbit Medicine, Faculty of Veterinary Medicine, University of Sadat City, Menoufiya, 32958 Egypt; 5 Botany and Microbiology Department, Faculty of Science, Cairo University, Giza, Egypt; 6 Biotechnology Department, Faculty of Science, Cairo University, Giza, Egypt

**Keywords:** Infectious bursal disease virus, IBDV, Phylogenetic analysis, Very virulent IBDV, Variant IBDV

## Abstract

Infectious bursal disease virus (IBDV) induces severe immunosuppression in chickens, leading to significant economic losses in the global poultry industry. This study investigated 52 chicken flocks, including commercial broilers, layers, and baladi, from various Egyptian governorates in 2023. These flocks exhibited symptoms of depression, along with kidney and bursa lesions, indicative of IBDV infection. Pooled Bursal homogenates were tested using RT-PCR with VP2-specific primers, revealing that 20 flocks tested positive for IBDV. Six representative samples were selected from 20 positive flocks for isolation in embryonated chicken eggs. The embryonic lesions observed included haemorrhage, skull swelling, and liver necrosis with a pale-yellow appearance, in addition to congestion and thickening in the chorioallantoic membrane (CAM). Partial amplification of the VP2 gene from the harvested embryo suspensions of the six IBDV isolates was performed for sequencing. Phylogenetic analysis of the sequences revealed that five IBDV isolates (VV4, VV5, VV6, VV10, and VV16) belonged to the very virulent strain group A3 cluster, whereas one isolate (VV2) clustered with Chinese Variant strains in the A2d group. Sequence analysis of the hypervariable region (HVR) of VP2 compared to that of Egypt-USC-IBD-1-2019 and vvIBDV/Beh21/Egypt/18 highly virulent IBDV strains revealed several amino acid mutations. The VP2 HVR of all isolates maintained the serine-rich heptapeptide sequence SWSASGS, which is adjacent to the major hydrophilic peak B and serves as a virulence marker. Histopathological examination revealed that bursae from chickens infected with vvIBDV exhibited marked interlobular oedema and lymphoid depletion. In contrast, bursae from chickens infected with Variant IBDV showed massive lymphoid depletion, with hyperplasia of the bursal capsule. These findings highlight the circulation of both virulent and Variant IBDV strains in Egyptian chicken flocks, complicating disease control. Consequently, there is a need to update vaccination programs and vaccine strains for IBDV in Egypt.

## Introduction

Infectious Bursal Disease Virus (IBDV) is a globally distributed pathogen that causes severe immunosuppression in chickens, leading to substantial economic losses in the poultry industry worldwide [3]. The virus is highly contagious, particularly among young chickens, and its resilience to environmental conditions and disinfectants makes it a formidable threat to poultry operations [31]. Once IBDV contaminates a poultry house, it can remain infective for at least four months, increasing the risk of recurrent infections in subsequent flocks, a concern faced by poultry farms around the world.

IBDV is distinguished by its bi-segmented, double-stranded RNA genome, which is prone to high mutation rates due to the error-prone nature of its RNA-dependent RNA polymerase (RdRp) [26]. These mutations, combined with recombination events, have led to the emergence of multiple IBDV strains, including classical IBDV (cIBDV), variant IBDV (VarIBDV), very virulent IBDV (vvIBDV), attenuated IBDV, and more recently, novel variant IBDV (nVarIBDV) [32]. Serotype I is the primary cause of disease in chickens, while serotype II, isolated from turkeys, does not induce illness in chickens (McFerran et al., 1980). The virus infects chickens of all breeds, and the genetic diversity among IBDV strains has complicated global control efforts, as mutant strains continue to spread and escape vaccine protection [30].

Globally, numerous studies have documented the emergence of variant IBDV isolates, emphasizing the virus’s ability to evolve and adapt to new environments and host immune responses [11, 14, 25]. In Asia, for instance, studies have identified significant mutations in the VP2 gene, which play a critical role in viral antigenicity and immune evasion [4]. Similar findings have been reported in Europe and North America, where variant strains have caused considerable challenges to existing vaccination programs [7, 10]. Recent research from regions such as China has identified mutations in variant IBDV strains that have led to severe outbreaks despite vaccination efforts [4]. These observations underscore the need for continuous surveillance of IBDV genetic variation across different regions, as well as regular updates to vaccine formulations to ensure broad protection.

The genetic diversity of IBDV has become a central challenge in controlling the virus. Mutations in the VP2 hypervariable region, responsible for coding the viral capsid protein, are associated with antigenic drift and immune escape, allowing the virus to evade neutralizing antibodies generated by commercial vaccines [6, 10]. The rapid evolution of these strains necessitates new methods for categorizing IBDV genotypes. Conventional classification systems, which are based on pathogenicity and antigenicity, are becoming increasingly inadequate due to the emergence of novel recombinant and mutant strains [6]. Recent advancements in genotyping systems have provided greater clarity in studying the molecular epidemiology of IBDV, helping to map the virus’s evolutionary trajectory [29].

Given the global nature of IBDV and its continued evolution, understanding the genetic variation of IBDV in specific regions is essential for designing effective control measures. In Egypt, where the poultry industry plays a critical role in the economy, IBDV poses a significant threat to both commercial and local chicken populations. While studies have confirmed the presence of both very virulent and variant IBDV strains in Egypt [14, 22], there has been limited research on the genetic dynamics of these strains in relation to global IBDV isolates. Therefore, our study seeks to isolate and characterize IBDV strains circulating in various Egyptian governorates, and to place these findings within the broader global context of IBDV evolution.

By comparing the genetic sequences of Egyptian IBDV isolates to those from other regions, including Asia, Europe, and North America, we aim to highlight the similarities and differences in IBDV strains across the globe. This will provide a clearer understanding of how variant strains in Egypt fit into the global IBDV landscape and will inform future vaccination strategies. The need to revise current vaccination programs in Egypt, as well as other IBDV-affected regions, is underscored by the rapid evolution of the virus and its capacity to evade immune responses. Our findings contribute to the growing body of international research on IBDV and provide essential data for the development of more effective global control strategies.

## Material and methods

### Sampling

Samples were collected from 52 commercial broilers, layers, and baladi chicken flocks with acute depression, nephritis, or bursal enlargement. During 2023, bursa of Fabricius of diseased and freshly dead chicks from 20 chicken farms in 6 governorates of Egypt (Alexandria, Giza, Beheira, Al-Qalubiya, Al-Mansoura, Al-Gharbia) were collected part kept in freezer for isolation and another part kept in buffered formalin for histopathology. For isolation, the bursae were homogenized with sterile sand and phosphate-buffered saline PBS containing 1000 μl/ml streptomycin and penicillin. Homogenized samples were centrifuged at 3000xg for 10 min. The supernatants were filtered using a 22 μl filter, and stored at −80 °C until use [20].

### Identification of IBDV using reverse transcription-polymerase chain reaction (RT-PCR)

RNA was extracted from the bursal tissue suspensions using the Gene JET Viral DNA/RNA Purification Kit (Thermo Scientific, Waltham, MA, USA) according to the manufacturer’s instructions. Two primers were designed in-house to amplify the 800 bp fragment of the IBDV VP2 gene by RT-PCR (forward primer: 5-CTGCAACAGCCAACATCAACG-3 and reverse primer: 5-CAAGACGGTCCCTCTCACT-3). cDNA was prepared using the Maxima H Minus Double-Stranded cDNA Synthesis Kit (Thermo Scientific, Waltham, MA, USA), according to the manufacturer’s instructions. PCR thermal cycler (StepOnePlus™ Real-Time PCR System, Applied Biosystems, Waltham, MA, USA) was used with the following cycling program: 95 °C for 5 min; 35 three-step cycles of 94 °C for 10s (denaturation), 52 °C for 20 s (annealing), and 72 °C for 50s; then 72 °C for 10 min (final extension). PCR products were electrophoresed against GeneRuler 100 bp Plus DNA Ladder (Thermo Scientific™, Waltham,) on a 1.5% agarose gel containing ethidium bromide at a final concentration of 0.5 μg/ml at 120V for 30 min. in 1 × TBE buffer [8].

### Virus isolation

Prepared bursal suspensions (0.2 ml) were injected into the chorioallantoic membrane (CAM) of 11–13-day old SPF-ECEs procured from the SPF farm at Koum Oshiem, Fayoum, Egypt. The inoculated eggs were incubated at 37°C for three–five days, then chilled at 4 °C overnight. For harvesting, CAM was obtained, washed three times with sterile PBS, and collected in a Falcon tube. Each sample was subsequently subjected to sonication for 1 min at water bath sonicator [19].

### Sequencing and sequence analysis of VP2 of IBDV

Same primers and conditions were used in RT-PCR with the RNA extracted from CAM of six representative IBDV isolates. Bands with the specific size were excised from the gel and purified by using the QIAquick Gel Extraction Kit (Qiagen, Hilden, Germany). The purified PCR products were sequenced with the ABI PRISM ® Big Dye TM Terminators v3.1 Cycle Sequencing Kit (Applied Biosystems, Foster City, CA, USA) and an ABI PRISM® 3130 genetic analyser with 80 cm capillaries (Applied Biosystems). The sequences were edited in SeqScape Software Version 2.5 (Applied Biosystems), and consensus sequence assembly and alignment trimming were performed using the Lasergene DNASTAR suite of programs (DNASTAR Inc., Madison, WI, USA) using the Clustal W method. According to [24], sequence alignment, identity, divergence, and phylogram were generated using the DNAstar-MegAlign program. Egyptian IBD viruses and other international reference strains were retrieved from NCBI, as were infectious bursal disease viruses from the National Centre for Biotechnology Information (NCBI) (http://www.ncbi.nlm.nih.gov).

### Histopathology

Bursas were collected from 3 to 5 birds from each flock and fixed in 10% neutral buffered formalin. The tissues were processed, and 4 μm-thick sections were cut from the paraffin-embedded tissue blocks. These sections were stained with hematoxylin and eosin, as described by [23], for routine histopathological (HP) examination under a light microscope.

## Results

### Farm history and mortality rates of IBDV-affected chicken flocks

Chickens from the examined farms displayed varying degrees of hemorrhage in their breast and thigh muscles, along with swollen and hemorrhagic bursa. The mortality rates in the examined chicken flock ranged from 3 to 20%, calculated over a 7-day period following the onset of increased mortality. The results indicated that both the type and timing of vaccination, as well as the age and type of chicken, significantly influenced the severity of IBDV infection and mortality rates. Mortality rates ranged from 2 to 20%, with the highest mortality observed in flock VV7 (20%), and the lowest in flocks VV9 and VV8 (3%). The severity of the lesions ranged from mild ( +) to severe (+ + +), with the most severe lesions observed in flocks VV5, VV10, and VV11. Moreover, the type and timing of vaccinations varied across flocks, with different vaccines, such as Transmmune, Vaxxitek, D78, Winterfield 228, IBDL (Ceva), Bursin Plus, Innovax, Bursaplex, and Intermediate, used at different ages (Table 1).

**Table 1:** Farm History Mortality rates for IBDV including age, vaccination program, severity of PM lesions, and location

Flock code	Chicken type	Governate	Age (days)	Flock size	IBDV vaccination		Mortality (%)	Severity of lesions
					Age	Vaccine		
VV1	Broiler	Alexandria	24	5000	1 8	Transmmune Intermediate	10	+ +
VV2	Broiler	Giza	24	20,000	1	Vaxxitek	6	+
VV3	Broiler	Beheira	30	110,000	1 8	vaxxitek D78	3	+ +
VV4	Layers	Al-Qalyubia	22	8000	13	Winterfield 228	6	+ +
VV5	Layers	Al-Qalyubia	39	4600	14	IBDL (ceva)	6	+ + +
VV6	Broiler	Giza	31	149,500	1 13	vaxxitek Intermediate	3	+ +
VV7	Broiler	Al-Qalyubia	30	5000	14	Bursin plus	20	+ +
VV8	Broiler	Al-Qalyubia	34	10,500	1 15	Innovax D78	3	+ +
VV9	Broiler	Al-Qalyubia	24	11,000	1 12	Bursaplex Intermediate	2	+
VV10	Layers	Al-Qalyubia	42	4500	7 12	inactivated IBDL ceva	12	+ + +
VV11	Layers	Al-Qalyubia	30	24,000	8	Inactivated	8	+ + +
					12	IBDL		
					19	Winterfield 228		
VV12	Broiler	Alexandria	30	8000	12	Intermediate	8	+ +
VV13	Broiler	Al-Qalyubia	32	7000	1	Transmmune	6	+ +
VV14	Baladi	Al-Mansoura	36	10,000	12	Intermediate plus	7	+
VV15	Broiler	Al-Mansoura	28	9000	1 8	vaxxitek Intermediate	4	+ +
VV16	Baladi	Al-Gharbia	30	10,000	12	Intermediate plus	6	+ +
VV17	Baladi	Al-Mansoura	25	7000	12	Intermediate plus		+ +
VV18	Broiler	Al-Qalyubia	25	3500	12	Hot	8	+ +
VV19	Broiler	Giza	24	20,000	1	Vaxxitek	5	+
VV20	Broiler	Giza	24	20,000	1	Vaxxitek	9	+

*Severity: + indicates mild, +  + indicates moderate, +  +  + indicates severe

### Molecular screening of IBDV using RT-PCR

Molecular screening of 52 chicken farms using RT-PCR with VP2-specific primers revealed that 20 farms (38.4%) tested positive for Infectious Bursal Disease Virus (IBDV).

### IBDV isolation

**Fig. 1:**
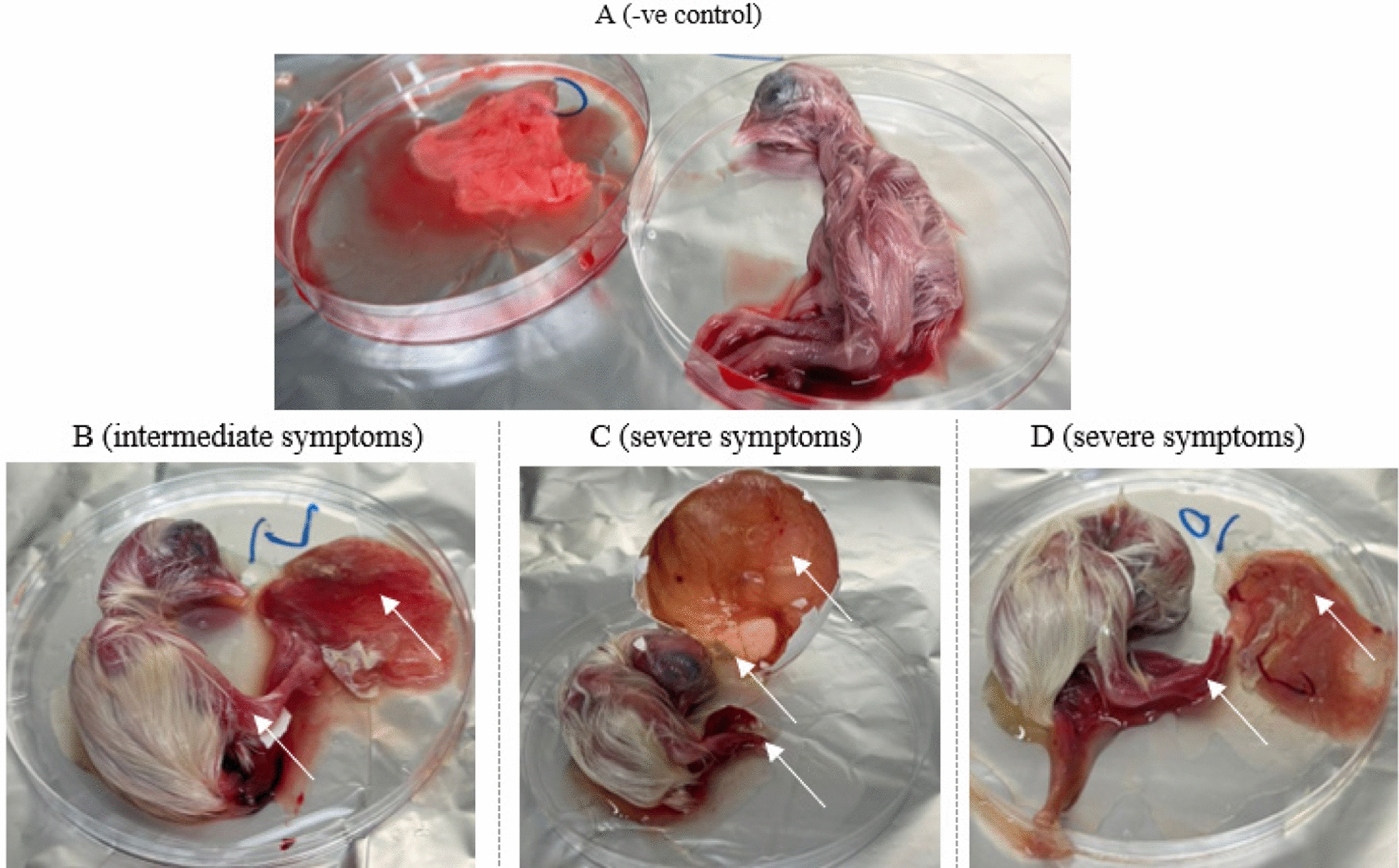
The harvested egg embryos showed hemorrhage, edema of the skull, harvested CAM became congested and thickened **A** negative control, **B** exhibited Intermediate symptoms on the embryo, **C** and **D** severe symptoms on the embryo

**Fig. 2:**
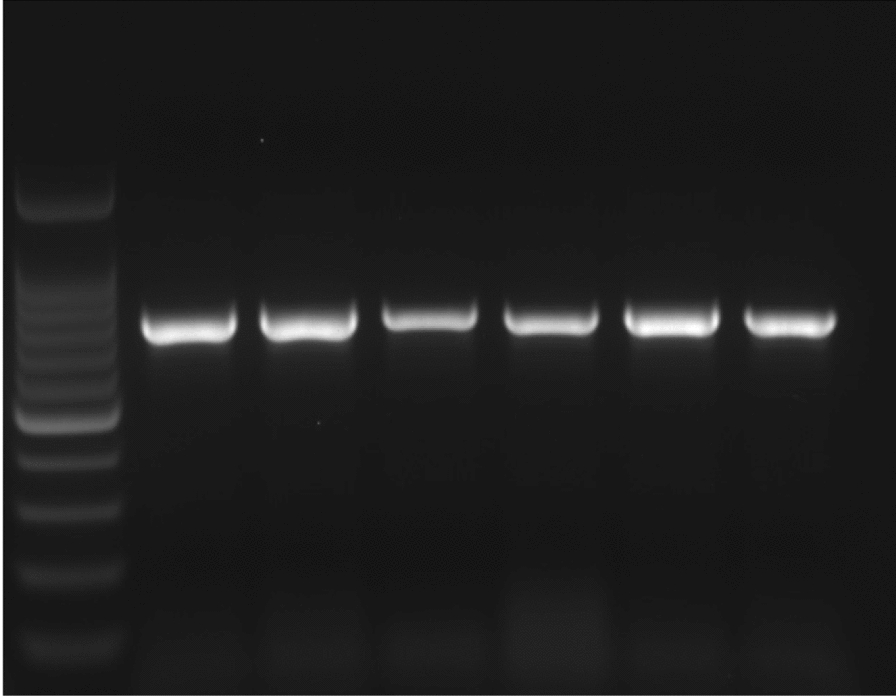
Gel electrophoresis for amplified VP2 bands by RT-PCR, the expected band size (800 bp). Lane 1 + ve control, Lanes 2–6 for IBDV strains isolated in this study

Six samples (VV2, VV4, VV5, VV6, VV10, and VV16) that give positive RT-PCR bands were inoculated into SPF-ECE via the chorioallantoic membrane (CAM) route at 11-day-old. Embryos showed signs suggestive of IBDV replication compared with the negative control. These signs included hemorrhage, skull swelling, and liver necrosis, which appeared pale yellow in color. Furthermore, the harvested CAMs were congested and thick (Fig. 1). CAM and liver from inoculated embryos were collected and processed for VP2 amplification by RT-PCR with successful amplification of 800 base pair bands (Fig. 2) that purified and sent for sequencing.

### Sequence analysis of the hyper Variable region of VP2

**Fig. 3:**
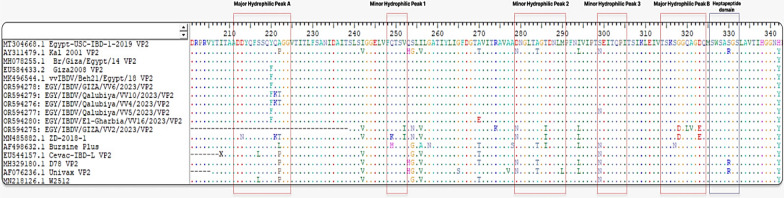
Deduced amino acid alignment of the VP2 hypervariable region from aa position 211 to 350 of IBDV strains. Dots indicate aa positions. Major and minor hydrophilic peaks are boxed with a red line

The hyper Variable region of VP2 was successfully amplified by RT-PCR from six selected IBDV isolates (VV2, VV4, VV5, VV6, VV10, and VV16). A 743-bp nucleotide sequence encoding 247 amino acids encompassing the region flanking the Hypervariable Region (HVR) of VP2 was characterized. For detailed analysis, a 420-bp segment encoding 140 amino acids within the VP2 HVR was examined (Fig. 3). All the identified and sequenced IBDV strains spanned nucleotide positions 625–1,044 and amino acids 211–350. Notable amino acid mutations were also observed in these isolates. VV5 exhibited S299N, VV10, VV4 showed Q221K and A222T, and VV16 had A270E. VV6 did not show any mutation. The VV2 isolate demonstrated multiple mutations (V252I, S254N, I256V, D279N, T286I, I294L, G318D, A321V, and D323E), indicating its relationship with the Chinese Variant strains. Despite these variations, all isolates retained the serine-rich heptapeptide sequence SWSASGS, a key virulence marker adjacent to major hydrophilic peak B.

**Fig. 4:**
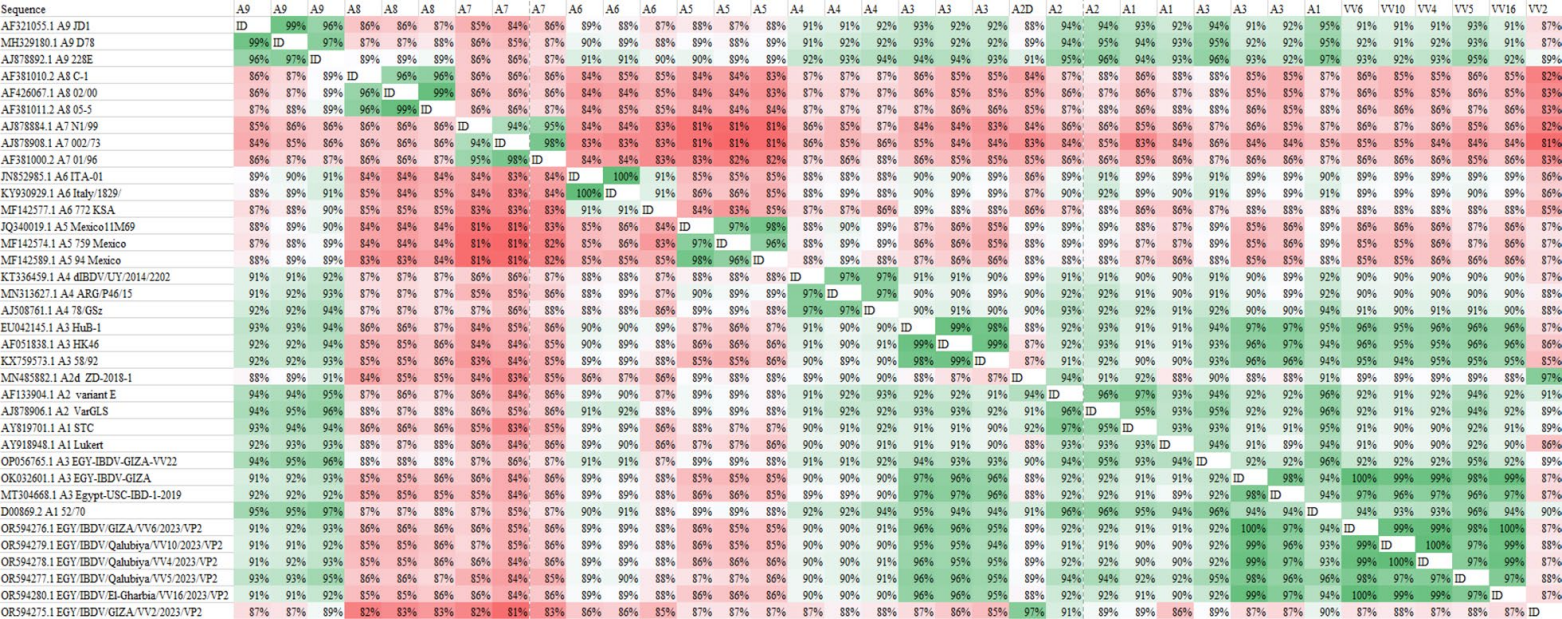
Identity Matrix between IBDV isolates and other Egyptian and representative reference strains

The identity matrix revealed that five isolates (VV4, VV5, VV6, VV10, and VV16) shared 94–100% identity with the A3 group reference strains, including Egypt-USC-IBD-1–2019, HuB-1, HK46, and 58/92. In contrast, the VV2 isolate showed 97% identity to the Chinese Variant, suggesting a close relationship with ZD-2018–1. All six isolates showed 90–94% identity with vaccine strain 57/70 in the A1 group (Fig. 4). (Table 2) contain a comprehensive illustration of amino acid mutations in VP2 compared to other isolates, as per the findings of a recent study [7].

**Table 2:** Amino acids mutation of VP2 for IBDv compared with isolates according to [7]

aa	Function	v v 4	v v 5	v v 6	vv 10	vv 16	v v 2	IB D- 1	MK496544.1 Beh21/Egypt/18	AF49 8632.1 Bursin e Plus	EU54 4157.1 Cevac -IBD- L	MN48 5882.1 ZD- 2018–1
213	Immune escape	D	D	D	D	D		D	D	D	D	N
219	Virus assembly	Q	Q	Q	Q	Q		Q	Q	Q	Q	Q
221	Immune escape	K	Q	Q	K	Q		Q	Q	Q	Q	K
222	Immune escape; Virus replication and virulence-related	T	A	A	T	A		A	A	L	P	T
223	Immune escape	G	G	G	G	G		G	G	G	G	G
234- 236	Intermolecular interactions	I D A	I D A	I D A	ID A	ID A		ID A	IDA	IDA	IDA	IDA
249	Immune escape; Virus replication and virulence-related	Q	Q	Q	Q	Q	Q	Q	Q	H	Q	K
253	Cellular adaptability; virulence-related	Q	Q	Q	Q	Q	Q	Q	Q	Q	Q	Q
254	Immune escape	S	S	S	S	S	N	S	S	G	G	N
256	Virus replication and virulence-related	I	I	I	I	I	V	I	I	A	V	V
270	attenuation	A	A	A	A	E	A	A	A	T	T	A
279	Cellular adaptability	D	D	D	D	D	N	D	D	D	D	N
284	Cellular adaptability	A	A	A	A	A	A	A	A	T	A	A
286	Immune escape	T	T	T	T	T	S	T	T	I	T	I
299	Cellular adaptability	S	N	S	S	S	E	S	S	N	N	S
318	Immune escape	G	G	G	G	G	D	G	G	G	G	D
321	Immune escape; virulence-related	A	A	A	A	A	V	A	A	A	A	A
323	Immune escape	D	D	D	D	D	E	D	D	D	D	E
324	Immune escape; virus assembly	Q	Q	Q	Q	Q	Q	Q	Q	Q	Q	Q

IBD-1: MT304668.1 Egypt-USC-IBD-1–2019.

### Phylogenetic analyses for HVR of VP2

**Fig. 5:**
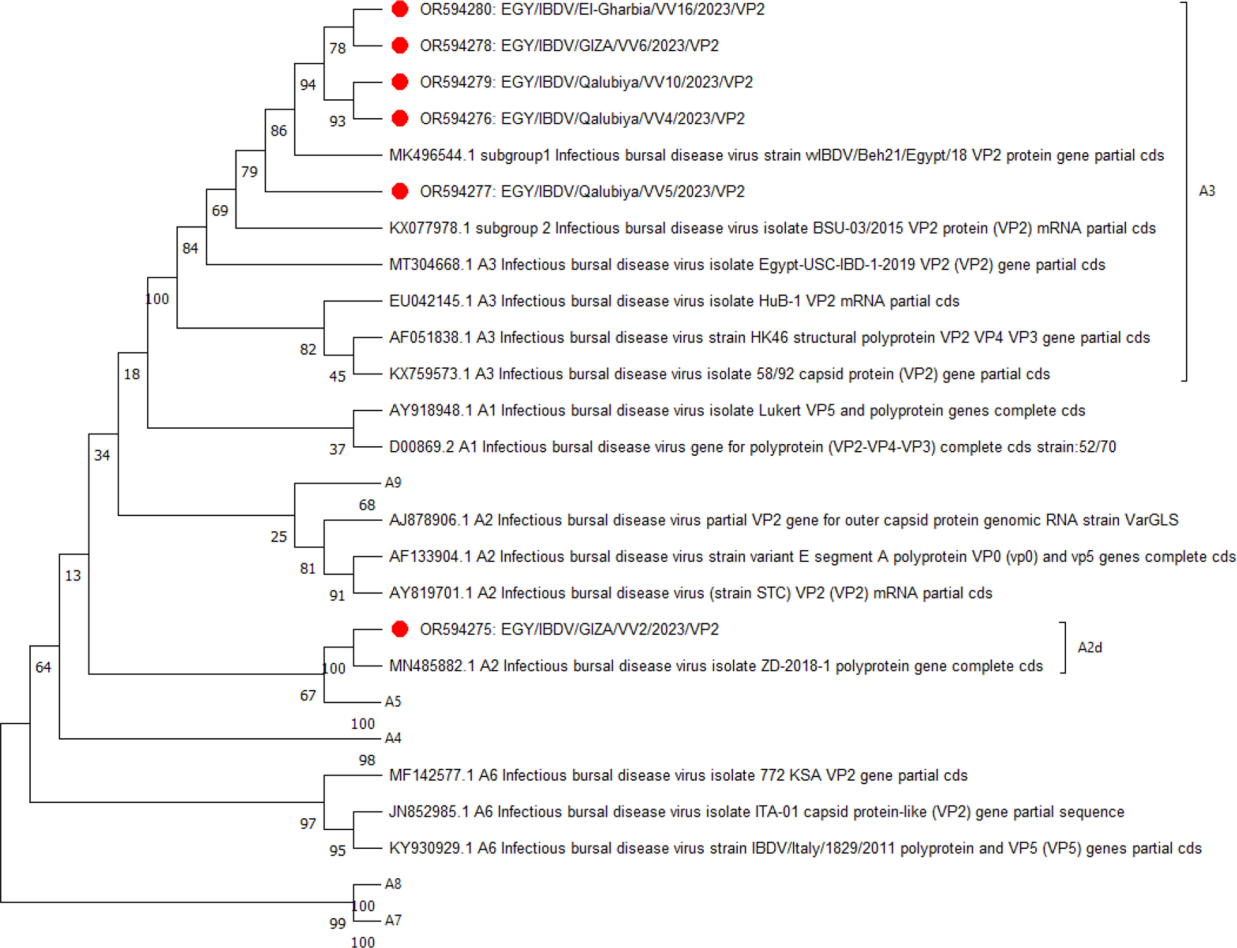
based on the nucleotide sequences by MAGE7.0. The phylogenetic tree represented the nucleotide sequences of VP2. The neighbour-joining phylogenetic trees were constructed using the Maximum Composite Likelihood model with 1000 bootstrap replicates

A phylogenetic tree constructed from the aligned HVR sequences revealed the formation of nine major clusters corresponding to the proposed genogroups. Among these, five isolates, EGY/IBDV/Qalubiya/VV4/2023,EGY/IBDV/Qalubiya/VV5/2023,EGY/IBDV/Qalubiya/VV10/2023, EGY/IBDV/El-Gharbia/VV16/2023, and EGY/IBDV/GIZA/VV6/2023, clustered within the A3 group of very virulent IBDV strains, as classified by [28]. This A3 cluster included strains commonly found globally, such as HuB-1 from Europe, HK46 from Asia, and Egypt-USC-IBD-1-2019 from the Middle East (Fig. 5). Notably, the isolate EGY/IBDV/GIZA/VV2/2023 was distinct, clustering with Chinese Variant strains within the A2d group.

### Histopathology

**Fig. 6:**
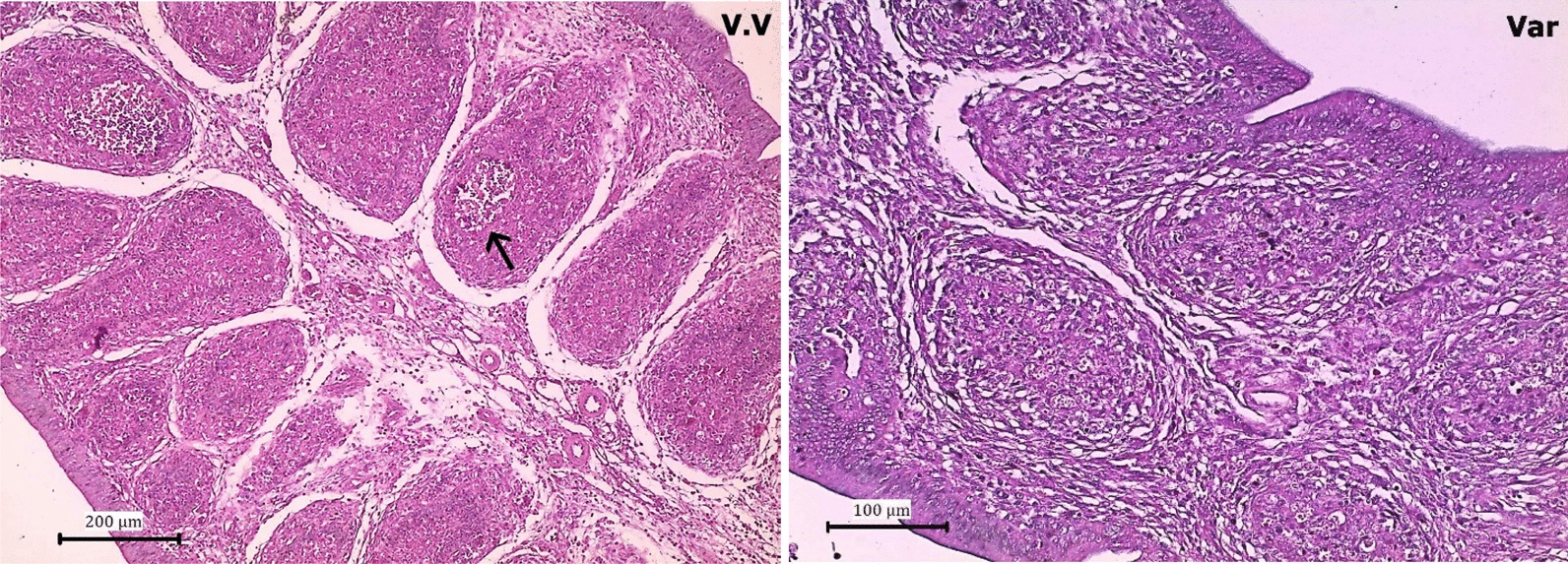
Photomicrograph of Bursa (VV): very virulent IBDV, bursa showed marked interlobular edema and lymphoid depletion (black arrow). (Var): Variant IBDV bursa showed massive lymphoid depletion with hyperplasia of bursal capsule

Histopathological analysis of bursae from chickens infected with very virulent IBDV (vvIBDV) demonstrated severe pathological changes. Specifically, the bursae exhibited marked interlobular edema, characterized by swelling between lobules and substantial lymphoid depletion, indicating a significant loss of lymphoid tissue. Lymphoid depletion disrupts the normal structure and function of the bursa, which is crucial for immune development in chickens. In contrast, the bursae from chickens infected with Variant IBDV showed a different pattern of pathology. Massive lymphoid depletion similar to that observed in vvIBDV infections was observed; however, this was accompanied by hyperplasia of the bursal capsule. Hyperplasia refers to an abnormal increase in the number of cells within the bursal capsule, which results in the thickening and enlargement of the capsule (Fig. 6).

## Discussion

Infectious Bursal Disease Virus (IBDV) remains a major threat to poultry health and productivity worldwide, including in Egypt, a leading poultry-producing country. Our study identified and characterized IBDV strains circulating in Egyptian chicken flocks, including both very virulent and variant strains. Detection of variant IBDV genogroup 2 in Argentenia in 2022 which showed increased evolution rate of 1.74 × 10^–3^ substitutions/site/year, while previously the country recorded only genogroup 4 till 2014 [11]. These findings align with global reports highlighting the increasing prevalence of variant IBDV strains and their ability to evade existing vaccines, making disease control a significant challenge across regions such as Asia, Europe, Africa and North America.

This study identified and characterized IBDV strains using RT-PCR, a highly sensitive method for detecting the virus [18]. Of the 52 field samples tested, 20 samples tested positive for IBDV. Seven flocks were tested positive from 24 tested flocks, two of them represent vvIBDV and 5 were related to novel variant IBDVs (A2dB1b) [14]. Another recent study revealed that 15 tested broiler flocks have only novel variant IBDVs (A2dB1b) while vvIBDV not detected [22].

Post-mortem examinations revealed a range of mild to severe lesions, including congestion and hemorrhage in the thigh muscles and edematous, hemorrhagic bursae. The observed clinical signs vary according to viral virulence, bird age, and immune status [21], highly virulent strains are known to cause more severe pathogenesis than less virulent ones [2].

The genetic diversity observed in our Egyptian IBDV isolates is consistent with reports from other regions, emphasizing the global nature of IBDV evolution. Studies from Asia, Europe, and North America reveal similar patterns of genetic drift and antigenic variation [5, 12]. Evolution of the antigenic variant IBDV (avIBDV) in South Korea with co-circulation of two sub-groups is recorded [25], this underlining the importance of international cooperation in monitoring and combating IBDV. This is essential to ensure that vaccination programs are continuously updated to reflect the latest genetic trends.

Our phylogenetic analysis revealed that five of the IBDV isolates (VV4, VV5, VV10, VV16, and VV6) clustered within the very virulent strain group (A3 cluster), consistent with findings from Europe and Asia [14]. The VV2 isolate, notably linked to Chinese variant strains, exhibited multiple amino acid mutations (e.g., V252I, T286I, and D323E), as previously reported in China [27]. This close phylogenetic relationship underscores the global spread of variant strains and their adaptive mutations across regions.

Amino acid mutations in the VP2 hypervariable region, particularly at positions such as 254S, 270A, and 286I, have been implicated in antigenic drift and immune escape in multiple regions [8]. Similar mutations were detected in our Egyptian isolates, suggesting that the virus’s ability to evade host immunity is a consistent evolutionary strategy across global poultry populations [9, 15]. The continuous evolution of IBDV strains globally has necessitated the development of new vaccines, as traditional strains no longer provide adequate protection [8]. For instance, in China, novel variant strains have caused severe damage to the Bursa of Fabricius in vaccinated flocks, despite pre-existing immunity to classical IBDV strains [27]. Our findings, particularly the isolation of Chinese-like variant strains in Egypt, suggest that vaccine programs in Egypt must be re-evaluated and updated to provide protection against such evolving variants. Egypt's poultry industry must adopt a similarly adaptive approach to those seen in China and other countries.

Our molecular analysis identified significant amino acid substitutions within the VP2 hypervariable region, notably S299N in isolate VV5 and D279N, G318D, and A321V in VV2. These mutations align with those found in variant strains isolated in Asia [13]. This genetic diversity is crucial to the virus’s ability to persist in poultry populations, as antigenic shifts allow IBDV to evade immune responses, even in previously vaccinated birds [10]. Our analysis also identified key mutations, such as S299N and Q221K, which are linked to antigenic variation, these mutations were not observed in previous studies by [14] and [22]. While, these mutations were consistent with those observed in global studies of variant strains [1, 17], further support the hypothesis that these mutations enhance viral evasion of host immune responses [27]. This emphasizes the necessity of global surveillance to track the emergence and spread of these variants.

The mutations identified in the VP2 hypervariable region of the Egyptian isolates have significant implications for vaccine efficacy. Globally, studies have documented similar mutations that result in antigenic drift, allowing variant strains to escape the immunity conferred by existing vaccines. The presence of these mutations in our isolates, particularly those such as 254S, 270A, and 286I, suggests that the virus is undergoing antigenic drift in Egypt, similar to patterns observed in Asia and Europe. These changes undermine the effectiveness of traditional vaccines, further complicating efforts to control the disease in Egypt and globally [1, 17, 27].

Moreover, our study’s results indicate that the evolutionary dynamics of IBDV in Egypt may mirror those observed globally, where selective immune pressures, driven by vaccination, are shaping viral mutations. For example, the presence of the A321V mutation in Egyptian isolates suggests that while this mutation may reduce viral pathogenicity in some contexts, it may still confer advantages in regions with high vaccine coverage, enabling the virus to persist in vaccinated flocks [14].

Histopathological analyses of bursa samples revealed distinct tissue damage patterns between chickens infected with very virulent (vvIBDV) and variant IBDV strains. Chickens infected with vvIBDV exhibited severe interlobular edema and lymphoid depletion, indicative of a profound immune disruption, which has been similarly observed in global outbreaks of very virulent IBDV strains [16]. In contrast, birds infected with variant strains demonstrated massive lymphoid depletion accompanied by hyperplasia of the bursal capsule, a compensatory mechanism that may reflect different pathogenic strategies employed by variant strains. These findings are consistent with those of [22], who reported similar pathological outcomes in variant IBDV-infected flocks in Egypt.

These findings contribute to the broader global understanding of IBDV’s genetic diversity and evolutionary dynamics. The rapid evolution of IBDV strains in Egypt, as demonstrated by our sequencing analysis, is consistent with global trends of antigenic drift and immune evasion. This highlights the need for continuous surveillance of IBDV mutations not only in Egypt but also worldwide, to ensure that vaccination programs remain effective. Moving forward, it is imperative that local vaccination programs in Egypt be adapted to account for the variant strains detected, aligning with global recommendations for the development of broad-spectrum vaccines capable of addressing the genetic diversity of IBDV.

In conclusion, this study provides critical insights into the genetic diversity and antigenic variation of IBDV in Egypt, positioning these findings within the broader global context of IBDV evolution. By identifying key mutations that mirror those found in other regions, we emphasize the importance of revising vaccination strategies to ensure protection against emerging variant strains. Our work adds to the growing body of evidence that IBDV remains a rapidly evolving pathogen, requiring global cooperation in surveillance and control efforts to mitigate its impact on the poultry industry.

## Conclusion

This study successfully characterized five very virulent IBDV (vvIBDV) strains and one Chinese Variant strain from field cases of infectious bursal disease (IBD) in Egypt in 2023. Genotyping revealed that these strains exhibited progressive evolution and persisted in an Egyptian environment, indicating an ongoing challenge with vvIBDV in the region. Our analysis demonstrated significant genetic diversity and pathogenic variability among IBDV strains in Egyptian chicken flocks. Molecular characterization of the VP2 hypervariable region (HVR) revealed critical genetic differences among the strains, impacting vaccine efficacy and disease management strategies. Additionally, the distinct histopathological profiles associated with different IBDV strains highlighted the need for tailored vaccination and control measures, which require continuous surveillance of circulating strains, regular updates to vaccination programs, and targeted control measures based on the characteristics of the prevalent strains. The identification of specific mutations within the VP2 HVR emphasized the virus's ongoing evolution, affecting its antigenicity and pathogenicity. In conclusion, the study's findings are pivotal for guiding vaccine development and understanding IBDV's adaptive mechanisms. The persistence of antigenically atypical strains in regions such as Egypt underscores the need for continuous monitoring and adaptation of vaccination strategies. Future research should focus on the evolutionary pressures driving these mutations and their impact on vaccine efficacy and viral control, to ensure effective strategies against evolving IBDV strains.

## Data Availability

No datasets were generated or analysed during the current study.
